# Single-cell transcriptome sequencing atlas of cassava tuberous root

**DOI:** 10.3389/fpls.2022.1053669

**Published:** 2023-01-04

**Authors:** Jinjia Song, Benji Fan, Xiaodie Shao, Yuwei Zang, Dayong Wang, Yi Min

**Affiliations:** ^1^ Department of Biotechnology, School of Life Sciences, Hainan University, Haikou, Hainan, China; ^2^ Laboratory of Biopharmaceuticals and Molecular Pharmacology, School of Pharmaceutical Sciences, Hainan University, Haikou, Hainan, China

**Keywords:** cassava, tuberous root, ScRNA-seq, casparian strip, differentiation trajectory

## Abstract

**Introduction:**

Single-cell transcriptome sequencing (ScRNA-seq) has emerged as an effective method for examining cell differentiation and development. In non-model plants, it hasn't been employed very much, especially in sink organs that are abundant in secondary metabolites.

**Results:**

In this study, we sequenced the single-cell transcriptomes at two developmental phases of cassava tuberous roots using the technology known as 10x Genomics (S1, S2). In total, 14,566 cells were grouped into 15 different cell types, primarily based on the marker genes of model plants known to exist. In the pseudotime study, the cell differentiation trajectory was defined, and the difference in gene expression between the two stages on the pseudotime axis was compared. The differentiation process of the vascular tissue and cerebral tissue was identified by the trajectory. We discovered the rare cell type known as the casparian strip via the use of up-regulated genes and pseudotime analysis, and we explained how it differentiates from endodermis. The successful creation of a protoplast isolation technique for organs rich in starch was also described in our study.

**Discussion:**

Together, we created the first high-resolution single-cell transcriptome atlas of cassava tuberous roots, which made significant advancements in our understanding of how these roots differentiate and develop.

## 1 Introduction

Cassava *(Manihot esculenta* Crantz) is the third most important source of calorie in Africa, Asia, and Latin America (after rice and maize) (Oliveira et al., 2014). Its starchy roots provide essential industrial materials and new sources of energy in addition to serving as a staple diet for millions of people in tropical and subtropical regions of the world (Li et al., 2017; Fathima et al., 2022). Cassava breeding is primarily focused on producing storage roots that are high yielding and of superior quality. Despite the importance of storage roots for the cassava, the origin of their development is poorly understood. Previous studies have described the anatomical process of differentiation of fibrous roots into storage roots by the section observation. (Indira and Kurian, 1977; Lowe et al., 1982; Wheatley et al., 2003). However, root-specific genes that can effectively distinguish emerging fibrous roots and storage roots have been identified by transcriptome analysis of roots collected before, during, and after root expansion, and it has been shown that storage roots contain internal channel structures that are continuous with stem secondary xylem, indicating that storage roots have a distinct rhizogenic process compared with fibrous roots (Carluccio et al., 2022). The anatomical structure of cassava tuberous roots is well established by 90 days after planting (Figueiredo et al., 2015). The mature tuberous root has five tissue layers visible on the transverse section. The epidermis, cambium, secondary phloem, parenchymatic cells, secondary xylem, and primary xylem are located from the outside in (Carvalho et al., 2002). Secondary growth is marked by the formation of vascular cambium, which produces secondary phloem tissue externally and secondary xylem tissue internally. After that, the secondary xylem differentiated into parenchyma cells, which expanded and accumulated starch (Lowe et al., 1982; Carvalho et al., 2018).

The occurrence of cassava tuberous root is a complicated process involving related endogenous and environmental signals, which requires various cell types to carry out different biological functions (Zierer et al., 2021). Currently, transcriptome research on the initiation and development of the cassava tuberous root mainly focused on the analysis of several metabolic pathways, including glycolysis/gluconeogenesis, amino acid metabolism, lipid metabolism (Sojikul et al., 2015; Rüscher et al., 2021). A significant advantage of a multi-omics analysis that integrates proteomics, transcriptomics, and metabolomics is the ability to identify highly dynamic and specific changes in how proteins or genes are expressed as roots develop (Ding et al., 2020; Utsumi et al., 2020). However, early transcriptome analysis treated the sample as a homogeneous material and averaged differences across hundreds or thousands of cells (Shaw et al., 2021). The patterns of gene expression at the cell level cannot be clearly revealed by a bulk gene expression profile from one tissue (Iqbal et al., 2020).

Single-cell transcriptome sequencing (scRNA-seq) has assisted researchers nowadays acquire critical new insights into cell-by-cell transcriptional analysis (Shaw et al., 2021). Since the first description of scRNA-seq (Tang et al., 2010), rapid advancements in single-cell transcriptome technology have made it an indispensable tool for research in many fields, including human, animal, and plant studies (Jovic et al., 2022). Since the preparation of plant single-cell suspension is tricker than that of animal due to the need to digest cell wall, the majority of current studies on plant single-cell primarily focus on model plants, such as *Arabidopsis thaliana* and *Oryza sativa L*. (rice) (Denyer et al., 2019; Ryu et al., 2019; Zhang et al., 2019; Liu et al., 2021b). The first scRNA-seq atlas was accomplished in Arabidopsis roots, demonstrating the viability of this technology in plants (Ryu et al., 2019). The single cell transcriptome technique can actually characterize common cell types and cell states, uncover rare cell types, and reveal, in particular, the relationship between cell differentiation and development among various cell types (Bawa et al., 2022). Since its invention, the single cell suspension technique has been used to study the genetics of maize, the regulation of tomato root initiation, and the differentiation trajectory of peanut leaf (Nelms and Walbot, 2019; Liu et al., 2021a; Xu et al., 2021; Omary et al., 2022). However, little is known about its application in tissues rich in secondary metabolites.

Early development is crucial in determining final tuber yields (Lowe et al., 1982). In the study, scRNA-seq was employed to explore the cell-by-cell heterogeneity of the cassava tuberous root at an early developmental stage, to track the differentiation trajectory of vascular tissue and cortical tissue and to examine functional enrichment among various tissues. It is projected that a high-resolution single-cell transcriptome atlas of the cassava tuberous root will be established, which will overcome the challenge of single-cell research in tissues rich in secondary metabolites and offer new insights into the tuberization and development of the root.

## 2 Materials and methods

### 2.1 Plant materials and growth conditions

The tuberous roots of cassava ‘South China 8’ (SC8) were used in this study. The materials were planted in Hainan University, Haikou City, Hainan Province, China (20°3΄47˝N, 110°19΄46˝E), at an elevation of 11 m, under field conditions. The climate is classified as tropical monsoon climate. The materials were planted on 2 and 3 April 2021 and collected on 2 July of the same year.

### 2.2 Preparation of tuberous root samples for ScRNA-seq

After collection, the tuberous roots were brushed and rinsed with water to move surface impurities and part of brown periderm. Fresh tuberous roots with the diameter of 0.5 cm (S1) and 0.75 cm (S2) were rapidly cut into small pieces and immersed in enzyme solution (10 mM MES, pH 5.7, 0.5 M mannitol, 2 mM KCl, 1.5% (w/v) Cellulose R10, 0.75% (w/v) Macerozyme, 0.1% (w/v) Pectinase, 1% (w/v) Cellulose RS, 10 mM CaCl_2_, 0.5% (w/v) BSA) at vacuum pump at room temperature for 10 min. Then the mixture was placed for 2-3 hours at 30°C and 65 rpm in dark and beat upon every 30 min to make the cell wall completely hydrolyzed and fully release the cells. The released protoplasts were filtered through 70 μm cell strainer, and rinsed with W5 (154 mM NaCl, 125 mM CaCl_2_, 5 mM KCl, 5 mM glucose, 1.5 mM NaCl, pH 5.6). The filtrate was centrifuged at 100 g at 4°C for 10 min. The precipitate was resuspended by adding WB (0.4 M mannitol, 0.5% BSA) and then centrifuged at 150 g at 4°C for 4 min. The precipitate was filtered again through 40 μm cell strainer. The process of centrifugation and resuspension mentioned above were repeated for protoplast solution. The oversized protoplasts and impurities were successfully filtered out, and a pure protoplast suspension was obtained. The protoplast viability was determined by trypan blue staining. The ratio of viable cells to total cells for S1 and S2 was >85% to meet the sequencing requirements. The concentration of protoplasts in each sample was counted by Countess^®^ II Automated Cell Counter (Thermofisher) and adjusted to 1000~2000 cells/μL.

### 2.3 ScRNA-seq library construction and sequencing

The single-cell suspensions of tuberous roots were loaded on a 10× Genomics GemCode Single-cell instrument to generate single-cell gel beads in emulsion (GEMs). The scRNA-seq library was prepared using the Chromium Next GEM Single Cell 3’ Reagent Kits v3.1 (10× Genomics, PN-1000121). The library was sequenced by Illumina sequencer NovaSeq 6000 using 150 bp paired-end kit. The raw scRNA-seq dataset was comprised of Read1, Read2, and i7 index read.

### 2.4 Cell clustering and identification of marker genes

The cells by gene matrices for each sample were individually imported to Seurat (version 3.1.1) for downstream analysis. Cells with unusually high number of UMIs (≥8000) or mitochondrial gene percent (≥10%) were filtered out. We also excluded cells with less than 540 or more than 5900 genes detected. Each individual dataset was scaled and normalized by the NormalizeData functions. The top 2000 highly variable genes were used for Principal Component Analysis (PCA) dimensionality reduction. The top 20 Principal Components (PCs) were selected according to the bending map of PCA, and the resolution parameter 0.3 was used for clustering (Liu et al., 2021b). Uniform Manifold Approximation and Projection (UMAP) (Becht et al., 2019) and t-Distributed Stochastic Neighbor Embedding (t-SNE) (Van der Maaten and Hinton, 2008) made clustering results visualized and easy to explore.

To determine the cell types of cassava tuberous root, marker genes were selected to identify 19 cell clusters. By homologous alignment of the genes of *Arabidopsis thaliana* and *Oryza sativa*, cell-specific marker genes were obtained. These marker genes were used for cell type annotation.

### 2.5 Pseudo time analysis

To observe the differentiation relationships among different cell cluster, we used Monocle (version 2.0) to construct its single cell pseudotime differentiation trajectory. To understand the key metabolic pathways related to the development and differentiation of vascular tissue, we performed pseudo-time-axis differential gene analysis of xylem, phloem and procambium. The genes with the top 100 P values in the branch of pseudo-time-axis were preserved and analyzed for functional enrichment, respectively. Subsequently, we drawn heat mapped of genes that are included in the significantly enriched pathways.

## 3 Results

### 3.1 ScRNA-seq and identification of cassava tuberous root cell clusters

**Figure 1:**
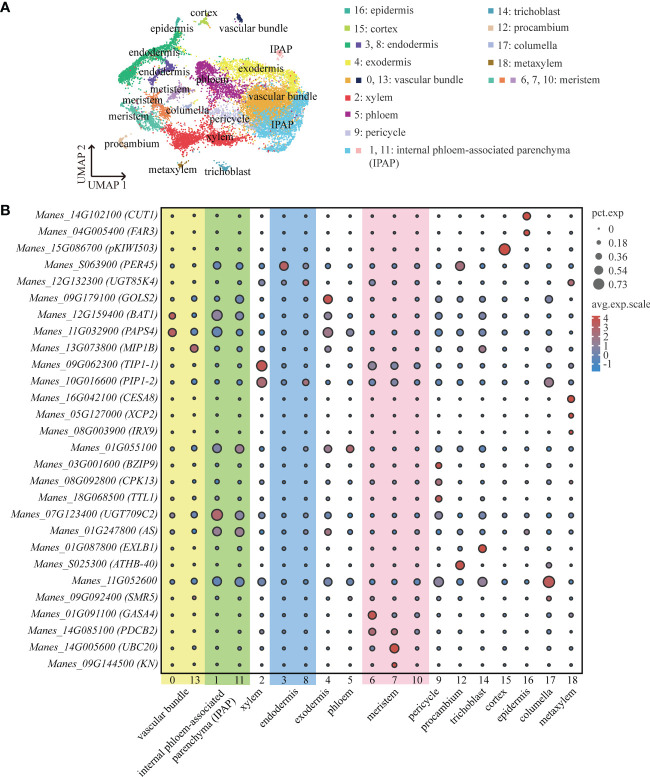
ScRNA-Seq and Identification of Cassava Cell Clusters. **(A)** Umap visualization of each cluster in the cassava tuberous root. Each dot indicates a single cell. Each color indicates a cell cluster. **(B)** Expression of genes associated with each cluster’s cell type. Dot size indicates the percentage of cells in the cluster that are expressing a particular gene, and color denotes expression across the cells in that cluster. *PEROXIDASE 45 (PER45)* was enriched in endodermis, *GALACTINOL SYNTHASE 2 (GolS2)* was enriched in exodermis, and *3-KETOACYL-CoA SYNTHASE 6 (CUT1)* and *FATTY ACYL-CoA REDUCTASE 3 (FAR3)* were enriched in epidermis. *CELLULOSE SYNTHASE A CATALYTIC SUBUNIT 8 (CESA8)*, *XYLEM CYSTEINE PEPTIDASE 2 (XCP2)* and *PROTEIN IRREGULAR XYLEM 9 (IRX9)* were enriched in metaxylem, *PROTEIN SIAMESE-RELATED 5 (SMR5)* was enriched in columella, *B-BOX DOMAIN PROTEIN 31 (MIP1B)*, *BIDIRECTIONAL AMINO-ACID TRANSPORTER 1 (BAT1)* and *NUCLEAR POLY(A) POLYMERASE 4 (PAPS4)* were enriched in vascular bundle, *CALCIUM-DEPENDENT PROTEIN KINASE 13 (CPK13)*, *BASIC LEUCINE ZIPPER 9 (BZIP9)* and *TETRATRICOPEPTIDE REPEAT THIOREDOXIN-LIKE 1 (TTL1)* were enriched in pericycle, and*7-DEOXYLOGANETIC ACID GLUCOSYLTRANSFERASE (UGT709C2)* and *ASYMMETRIC LEAVES (AS)* were enriched in internal phloem-associated parenchyma (IPAP). *TONOPLAST INTRINSIC PROTEIN 1-1 (TIP1-1)* and *PLASMA MEMBEANE INTRINSIC PROTEIN 1-2 (PIP1-2)* were enriched in xylem.

We performed high-throughput scRNA-seq for plant tissues from cassava tuberous roots with diameters of about 0.5 cm (S1) and 0.75 cm (S2), respectively. After preparing protoplast suspension and capturing single cells with 10×Genomics, we successfully detected 7511 cells at S1 and 7055 cells at S2. The average number of genes detected per cell was 1274 and 1209, respectively (**Supplemental Table 1**). PCA was used to project the data into linear dimensions. To effectively convert the high-dimensional data into the two-dimensional image, t-SNE or UMAP was used to further reduce the dimension of the cells. These unsupervised clustering analyses divided these cells into 19 cell clusters. The cell number of each cluster accounted for 0.3%-32.02% of the total cell number (**Figure 1A**; **Supplemental Table 2**).

To annotate the cell types to which each clusters belonged, we found about thirty orthologs of known marker genes which had been identified for cell types in model plants such as *Arabidopsis thaliana* and *Oryza sativa*. With respect to the cortical tissues, we selected *PEROXIDASE 45 (PER45)* for endodermis (Long et al., 2021); *GALACTINOL SYNTHASE 2 (GolS2)* for exodermis (Sprenger and Keller, 2000); *3-KETOACYL-CoA SYNTHASE 6 (CUT1)* and *FATTY ACYL-CoA REDUCTASE 3 (FAR3)* for epidermis (Hooker et al., 2002; Rowland et al., 2006). For the vascular tissues, we used *CELLULOSE SYNTHASE A CATALYTIC SUBUNIT 8 (CESA8)*, *XYLEM CYSTEINE PEPTIDASE 2 (XCP2)* and *PROTEIN IRREGULAR XYLEM 9 (IRX9)* for metaxylem (Turco et al., 2019; Zhang et al., 2019; Li et al., 2021); *PROTEIN SIAMESE-RELATED 5 (SMR5)* for columella (Yi et al., 2014); *B-BOX DOMAIN PROTEIN 31 (MIP1B)*, *BIDIRECTIONAL AMINO-ACID TRANSPORTER 1 (BAT1)* and *NUCLEAR POLY(A) POLYMERASE 4 (PAPS4)* for vascular bundle (Meeks et al., 2009; Choi et al., 2013; Graeff et al., 2016); *CALCIUM-DEPENDENT PROTEIN KINASE 13 (CPK13)*, *BASIC LEUCINE ZIPPER 9 (BZIP9)* and *TETRATRICOPEPTIDE REPEAT THIOREDOXIN-LIKE 1 (TTL1)* for pericycle (Saijo et al., 2001; Lakhssassi et al., 2012; Jean-Baptiste et al., 2019); *7-DEOXYLOGANETIC ACID GLUCOSYLTRANSFERASE (UGT709C2)* and *ASYMMETRIC LEAVES (AS)* for internal phloem-associated parenchyma (IPAP) (Song et al., 2012; Asada et al., 2013). Furthermore, it has been demonstrated that the members of the larger family of major intrinsic proteins (MIPs), *TONOPLAST INTRINSIC PROTEIN 1-1 (TIP1-1)* and *PLASMA MEMBEANE INTRINSIC PROTEIN 1-2 (PIP1-2)*, localize in conducting tissue and xylem parenchyma cells, respectively (Barrieu et al., 1998; Fetter et al., 2004). The vessels of xylem were contained in the conducting tissue (Qiang et al., 2013). Therefore, for the xylem, we used TIP1-1 and PIP1-2. The representative marker genes of meristem included *GIBBERELLIN-REGULATED PROTEIN 4 (GASA4)*, *PLASMODESMATA CALLOSE-BINDING PROTEIN 2 (PDCB2)*, *UBIQUITIN-CONJUGATING ENZYME E2 20 (UBC20)* and *SYNTAXIN-111 (KN)* (Criqui et al., 2002; Roxrud et al., 2007; Simpson et al., 2009; Wendrich et al., 2020). *HOMEOBOX-LEUCINE ZIPPER PROTEIN (ATHB-40)* is specifically expressed in procambium (Wendrich et al., 2020). As shown in the **Figure 1B**, according to the expression of marker genes in each cluster, we defined cluster 3 as endodermis, cluster 4 as exodermis, cluster 16 as epidermis, cluster 2 as xylem, cluster18 as metaxylem, cluster 17 as columella, cluster 13 and cluster 0 as vascular bundles, cluster 9 as pericycle, cluster 1 and cluster 11 as internal phloem-associated parenchyma, cluster 6 and cluster 7 as meristems and cluster 12 as procambium. In addition, by homology alignment with *Arabidopsis thaliana* and *Oryza sativa*, we defined cluster 15 as cortex and cluster 14 as trichoblast, given that *MERALLOTHIONEIN-LIKE PROTEIN TYPE 3 (pKIWI503)* was highly expressed in cluster 15 (Liu et al., 2021b) and *EXPANSIN-LIKE B1 (EXLB1)* was highly expressed in cluster 14 (Won et al., 2010). And *Manes_11G052600* was highly expressed in cluster 17, which further confirmed the fact that cluster 17 was columella (**Supplemental Table 5**) (Denyer et al., 2019; Ryu et al., 2019; Shahan et al., 2022). Through the venn diagram analysis of the significant up-regulated genes, we found that cluster 10 had a large number of mutual up-regulated genes shared by cluster 6 and cluster 7 that were considered as meristems (**Supplemental Figure 2D**). We further performed functional enrichment analysis on these three clusters and found that they were mainly enriched in the synthesis and metabolism of ribosome and the activities of structural molecular (**Supplemental Figure 2A-C**), indicating that cluster 10, the same as meristems, had active events of cell division. Therefore, the cluster 10 was defined as the meristem (**Supplemental Table 10**).

The combination of up-regulated genes analysis and pseudotime analysis was a good way to identify the clusters that were difficult to distinguish. *LINAMARIN SYNTHASE 1 (UGT85K4)*, the up-regulated gene of cluster 8, was highly expressed in cortical tissues (Kannangara et al., 2011) (**Figure 1B**). The results of pseudotime analysis showed that cluster 8 was in the same branch with endodermis (**Supplemental Figures 1A, B**). The *Manes_01G055100* (Avr9/Cf-9 rapidly elicited protein) is homologous to marker gene *AT1G52140* (Avr9/Cf-9 rapidly elicited protein) of *Arabidopsis* phloem (Wendrich et al., 2020), is highly expressed in cluster 5 (**Figure 1B**). By pseudotime analysis we found that cluster 5, in line with xylem, was differentiated from procambium (**Supplement Figures 1C, D**). It was consistent with the previous results on the differentiation relationship between phloem and procambium (Lucas et al., 2013). Therefore, we defined cluster 8 as endodermis and cluster 5 as phloem (**Supplemental Table 4**).

### 3.2 Differentiation trajectory of xylem and phloem

**Figure 2:**
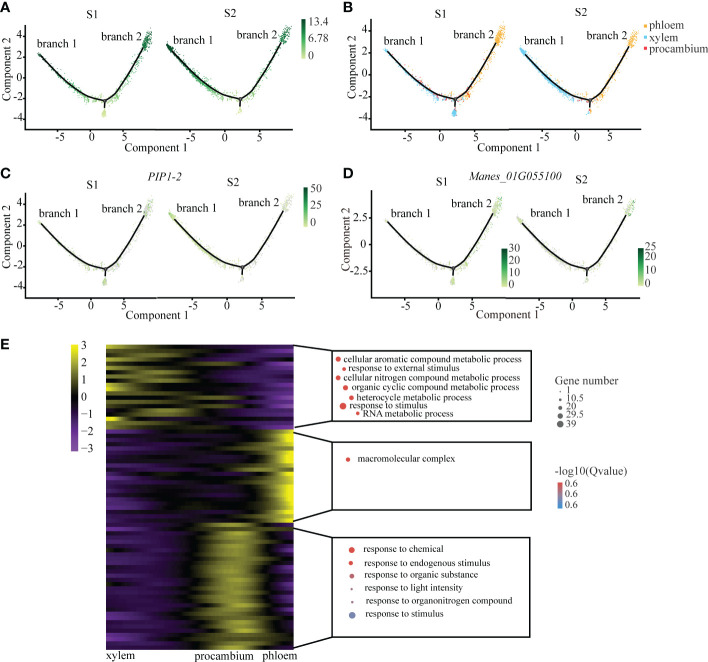
Differentiation Trajectory of the Xylem and Phloem. **(A)** Single-cell transcriptome data revealing the differentiation trajectory of xylem, phloem and procambium from S1 and S2 analyzed by Monocle 2. Each dot indicates a single cell. Different color on the dots indicates the pseudotime scores **(B)** Cell types labeled on the differentiation trajectory for S1 and S2, respectively. **(C, D)** Cell trajectory analysis of differentiation fate of xylem marker gene *PIP1-2* **(C)** and phloem marker gene *Manes_01G055100* **(D)** from S1 and S2. Each dot indicates a single cell. Color on the dots indicates the expression abundance in corresponding parts. **(E)** Heatmap showing the expression of the genes regulating significant enrichment function in three clusters across the pseudotime. Each row represents one gene. Scatter plots of representative GO terms for each cluster are shown on the right. The corresponding genes in the heatmap are listed in the supplementary table of CT roots.

We observed that xylem and phloem were differentiated from procambium, which is in keeping with the results of previous studies (Lucas et al., 2013). After a symbolic bifurcation point which marks developmental state transition, procambium cells were divided into two differentiated branches: one was xylem (branch 1), and the other was phloem (branch 2) (**Figure 2A**). From the cell trajectory diagram of differentiated fate, *PIP1-2*, the xylem marker gene, was highly expressed in the branch 1, while *Manes_01G055100* that is the phloem marker gene was highly expressed in the branch 2. The pseudotime score gradually increased along the branches (**Figures 2C, D**). In addition, the number of xylem cells at S2 was more than that at S1, and the number of phloem cells at S2 was less than that at S1 (**Figure 2B**). It is consistent with the fact that the cell number of xylem increase with cassava tuberous root enlargement (Figueiredo et al., 2015).

In order to explore the key pathways involved in the regulation of the vascular tissues of cassava tuberous root, we performed functional enrichment analysis of differential genes in xylem and phloem on the pseudotime axis. The results showed that xylem differential genes were mainly related to organic metabolism and response to external stimuli, suggesting that they may be involved in the process of starch accumulation and water transportation in xylem (Spicer and Groover, 2010). The phloem differential genes were mainly related to the regulation of macromolecule metabolism, which indicated that they may be involved in the transportation of products of photosynthesis in phloem (Fukuda and Ohashi-Ito, 2019). The differential genes of procambium were enriched in small molecular metabolic pathways, suggesting that it may be sensitive to plant hormones such as auxin (**Figure 2E**; **Supplemental Table 6**).

### 3.3 Differentiation trajectory of the cortical tissue

**Figure 3:**
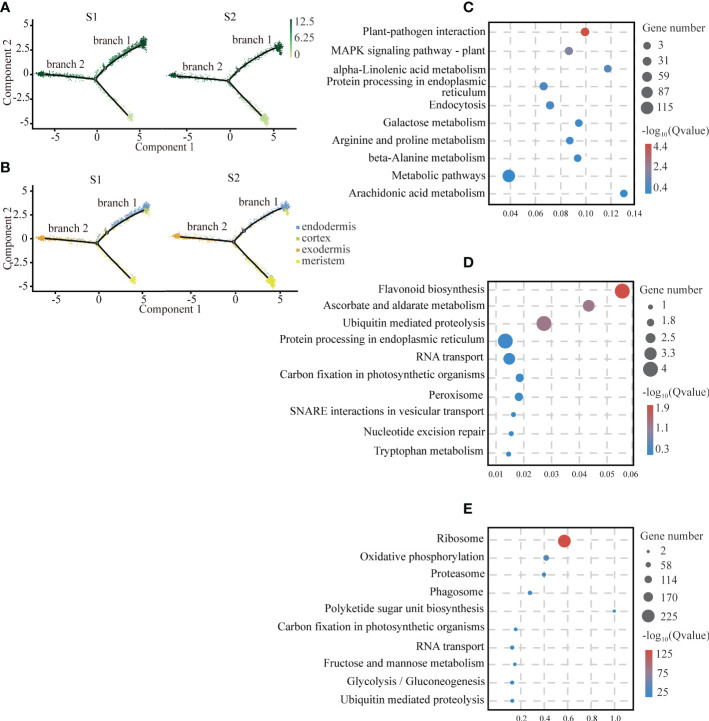
Differentiation Trajectory of Cartical Tissue. **(A)** Single-cell transcriptome data revealing the differentiation trajectory of cortical tissue and meristem from S1 and S2 analyzed by Monocle 2. Each dot indicates a single cell. Different color on the dots indicates the pseudotime scores. **(B)** Cell types labeled on the differentiation trajectory for S1 and S2, respectively. **(C–E)** Scatter plots of GO and KEGG enrichment analysis of up-regulated genes in exodermis **(C)**, endodermis **(D)** and meristem **(E)** cell types after the aggregation of S1 and S2.

In the plant root, the cortical tissue is the basic organization between epidermis and vascular bundle and plays an important role in the storage of carbohydrates and temporary metabolites. We observed that there was a differentiation relationship between cortical tissue and meristem of the cassava tuberous roots. In the cell trajectory diagram of the pseudotime analysis, meristem cells served as the starting branch of differentiation and then gradually differentiated into two branches: endodermal cells were in branch 1, exodermis cells were in branch 2, and cortex cells were scattered in branch 1 and branch 2 (**Figure 3A**). The number of cells of cortical tissue at S2 was significantly less than S1 (**Figure 3B**). It is consistent with the results that the cortical tissue became thinner with the tuberous root development (Figueiredo et al., 2015).

To provide insights into the biological functions of the cortical tissue and the meristem, we conducted functional enrichment analysis of them. We found that genes related to plant disease-resistant microorganisms and antioxidants were enriched in the cortical tissue, suggesting that it might be the tissue that provided biological protection for the tuberous root. The exodermis was mainly enriched with genes interacting with plant-pathogen interactions. Among these genes, we found several homologous genes of cyclic nucleotide-gated ion channels (CNGCs) (Leng et al., 1999) from animals, which penetrated K^+^ and Ca^2+^ in a cyclic nucleotide-dependent manner and had a strong selectivity for Na^+^ (Clough et al., 2000). At the same time, we also observed that many calcium-binding proteins and calcium-dependent proteins were enriched in this pathway. It indicated that they played an important role in resisting pathogen invasion (**Figure 3C**; **Supplemental Table 7**). The endodermis highly expressed with the genes related to flavonoid biosynthesis and ascorbate and aldarate metabolism, suggesting that these substances might be synthesized in the endodermis (**Figure 3D**; **Supplemental Table 8**). The genes that highly expressed in the meristem associated with the ribosomal activity and oxidative phosphorylation. The result was consistent with the fact that the meristem had strong mitotic activity (**Figure 3E**; **Supplemental Table 9**).

**Figure 4:**
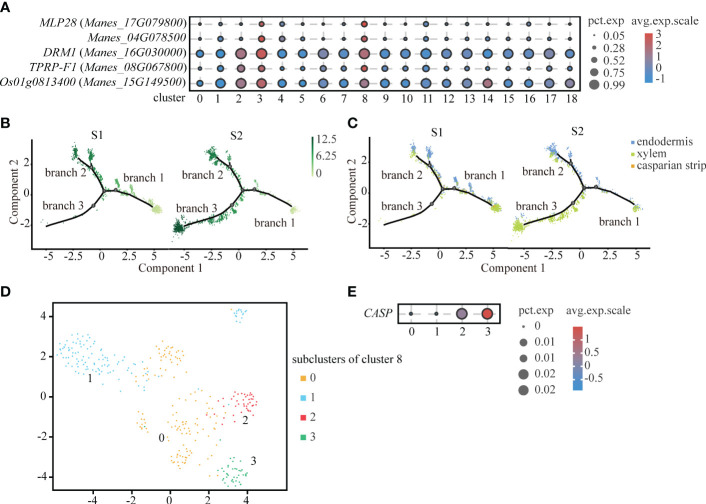
Differentiation Trajectory of Casparian strip. **(A)** Expression of significantly up-regulated genes in cluster 3 (endodermis) and cluster 8. Clusters 0 to 18 are classified according to cell-by-cell correlation. The cell types corresponding to each cluster are listed in the **Supplemental Table 2**. The dot size indicates the fraction of cells in each cluster expressing a given gene, and the color indicates the level of signification up-regulated genes exhibit the preferential expression in corresponding cluster. **(B)** Pseudotime analysis using monocle 2 for single-cell transcriptome in the endodermis, xylem and casparian strip revealing the differentiation trajectory. Each dot indicates a single cell. Different color on the dots indicates the pseudotime score. **(C)** Cell types labeled on the differentiation trajectory for S1 and S2, respectively. **(D)** UMAP exhibition of 4 subclusters after the subcluster analysis of cluster 8. Each dot indicates a single cell. **(E)** Expression abundance of 4 subcluster of cluster 8. The dot size indicates the fraction of cells in each cluster expression the given gene, and the color denotes the level of marker genes exhibit the preferential expression in corresponding cluster.

Interestingly, through the analysis of up-regulated genes, we found that cluster 8 and endodermis shared some significantly up-regulated genes, such as *MLP-like protein 28 (MLP28)*, *Manes_04G078500*, *Dormancy-associated protein 1 (DRM1)* and so on. By comparing the significantly up-regulated genes in cluster 8, we found that the significantly up-regulated genes of cluster 8, such as *36.4 kDa proline-rich protein (TPRP-F1)*, were also highly expressed in endodermis. And some significantly up-regulated genes of cluster 8, such as *ADP-ribosylation factor 1 (Os01g0813400)*, were expressed in xylem (**Figure 4A**). Therefore, we speculated that cluster 8 might be a type of cells in between endodermis and vascular tissue. As shown in the **Figures 4B, C**, cluster 8 is mainly distributed in branch 2. Endodermis is mainly distributed in branch 2 and xylem is mainly distributed in branch 3.

In plants, except for cells facing stem axis and the surface of root and stem, the endodermal cell wall is surrounded by lignified and suberized hydrophobic structure called casparian strip. Endodermis with casparian strip can regulate the water flow and mineral ionic equilibrium between external tissue and vascular bundle (Nagahashi et al., 1974). However, because the cell number of casparian strip is scarce, we divided cluster 8 into four sub-clusters (**Figure 4D**). As a rare cell type, the casparian strip was not readily detected by up-regulation of genes expression. *Casparian strip membrane domain protein (CASP)* is a specifically expressed gene in the casparian strip and plays an important role in the formation of the casparian strip (Roppolo et al., 2011). We found that *CASP* was relatively highly expressed in subcluster 3 (**Figure 4E**). Therefore, we defined subcluster 3 as the casparian strip.

## 4 Discussion

Preparation of plant single-cell suspensions is trickier than that of animal cells due to the need to digest cell wall. New challenges and new possibilities might emerge when applying this technology in starch-rich tissues, such as the influence of free starch on suspension preparation, the interference of free starch on sequencing results, and the acquisition of transcriptome information. Our results might provide some reliefs for these concerns. Firstly, most of the free starch particles could be removed by multiple washing in the preparation stage of protoplast suspension. Secondly, enough cells combined with enzymes and gel beads containing barcode information were wrapped into oil droplets to form gel beads in-emulsions (GEMs). If a small amount of free starch granules were wrapped in GEMs as cells, the obtained data would also be filtered out by Cell Ranger software and Seurat software in the data analysis stage. Thirdly, the number of filtered cells is still sufficient to obtain efficient data sets as most of the cells in cassava tuberous roots are less than 40 microns in diameter, even starch-rich cells can be detected by their pore size. Finally, the amplified products meet the sequencing standards, and the transcriptional information is normal, indicating that starch does not affect the reverse transcription results at the sequencing stage. Therefore, the application of single cell transcriptome in starch-rich plant tissues is feasible.

The cortex which is a ground tissue and lies between the epidermis and vascular tissue, provide support, and perform metabolic processes in plant root (Soukup et al., 2005). The cortex of Arabidopsis, which consists of a single layer of cells, is derived from cortex initial cells (Petricka et al., 2012). However, according to Figueiredo et al. (2015), cassava tuberous roots are the result of secondary growth, and the cortex only exists in the organs of primary growth or the early stages of secondary growth. Therefore, the term cortex should not be used to describe cassava tuberous roots. However, in this study, we identified a small number of cells of cortex at S1 and S2 of tuberous roots, accounting for 0.97% and 0.48% of the total cells, respectively. It fully demonstrated the advantages of single-cell transcriptome technology compared to section technology in observing clusters with rare number of cells.

The pseudotime analysis is not only a way to study cell differentiation trajectories, but also an approach to mine rare cell types. Since single-cell research relied on the number of captured cells, the identification of the cell clustering with a little number of cells existed obstacles. The casparian strip is deposited on the radial parts of cell wall of the endodermal cells (Chen et al., 2011) and acts as a barrier for the diffusion of material through the cell wall from cortical tissues to vascular tissues (Nagahashi et al., 1974). We observed that some up-regulated genes in cluster 8 were expressed in both endodermis and xylem, suggesting that cluster 8 might be associated with endodermis and xylem. Through pseudotime analysis, we further confirmed sub-cluster 3 of cluster 8 as the casparian strip. According to the pseudotime analysis in our results, the casparian strip was differentiated from endodermis, which was consistent with previous studies (Bonnett, 1969; Robards and Robb, 1972). Although pseudotime analysis did not reveal a differentiated relationship between the casparian strip and xylem, there were up-regulated genes related to lignification in both parts. This result is consistent with previous results that both the casparian strip and xylem were highly lignified tissues, containing a large amount of lignin and similar cell wall components (Zeier et al., 1999; Spicer and Groover, 2010).

In conclusion, we successfully mapped a single cell transcriptome atlas of starch-rich cassava tuberous root, observed the differentiation relationships among the cell clusters by pseudotime analysis, and identified the rare cell type, the casparian strip. This study laid a foundation for mining the development and genetic improvement of cassava tuberous root.

## Data availability statement

The original contributions presented in the study are publicly available. This data can be found here: NCBI, PRJNA895163, https://www.ncbi.nlm.nih.gov/bioproject/PRJNA895163.

## Author contributions

YM conceived the ideas and the overall study design. YZ collected the data. JS, BF, and XS analyzed the data and led the writing of the manuscript. DW revised the manuscript. All authors contributed critically to the drafts and gave final approval for publication.

## Supplementary material

Supplementary Figure 1 Identification of Cluster 5. **(A)** Single-cell Transcriptome data of endodermis, cluster 8 and phloem from S1 and S2 analyzed by Monocle 2, revealing the differentiation trajectory of them. Each dot indicates a single cell. Different color on the dots denotes the pseudotime score. **(B)** Cell types labeled on the differentiation trajectory for S1 and S2, respectively. **(C)** Single-cell Transcriptome data of xylem, cluster 5 and procambium from S1 and S2 analyzed by Monocle 2, revealing the differentiation trajectory of them. Each dot indicates a single cell. Color on the dots indicates the pseudotime score. **(D)** Cell types labeled on the differentiation trajectory of S1 and S2, respectively.

Supplementary Figure 2 Identification of Cluster 10. **(A–C)** Scatter plots of GO enrichment analysis of up-regulated genes for cluster 6 **(A)**, cluster 7 **(B)** and cluster 10 **(C)** cell types after the aggregation of S1 and S2. **(D)** Scatter plots of GO enrichment analysis of 56 up-regulated genes specific to cluster 10 cell types after the aggregation of S1 and S2.

Supplementary Figure 3 The Umap visualization of each cluster in the cassava tuberous root of S1 and S2, respectively. **(A)** Umap visualization of each cluster in the cassava tuberous root of S1 stage. Each dot indicates a single cell. Each color indicates a cell cluster. **(B)** Umap visualization of each cluster in the cassava tuberous root of S2 stage. Each dot indicates a single cell. Each color indicates a cell cluster. **(C)** The cross section of the Cassava tuberous root.

Supplementary Tables 1–10 Statiatical data of scRNA-seq of cassava tuberous root.
