# Supplementary figures and images for: Single-cell transcriptome sequencing atlas of cassava tuberous root
Source: Front Plant Sci. 2023 Jan 4;13:1053669. doi: 10.3389/fpls.2022.1053669 (PMC9848496; doi:10.3389/fpls.2022.1053669)

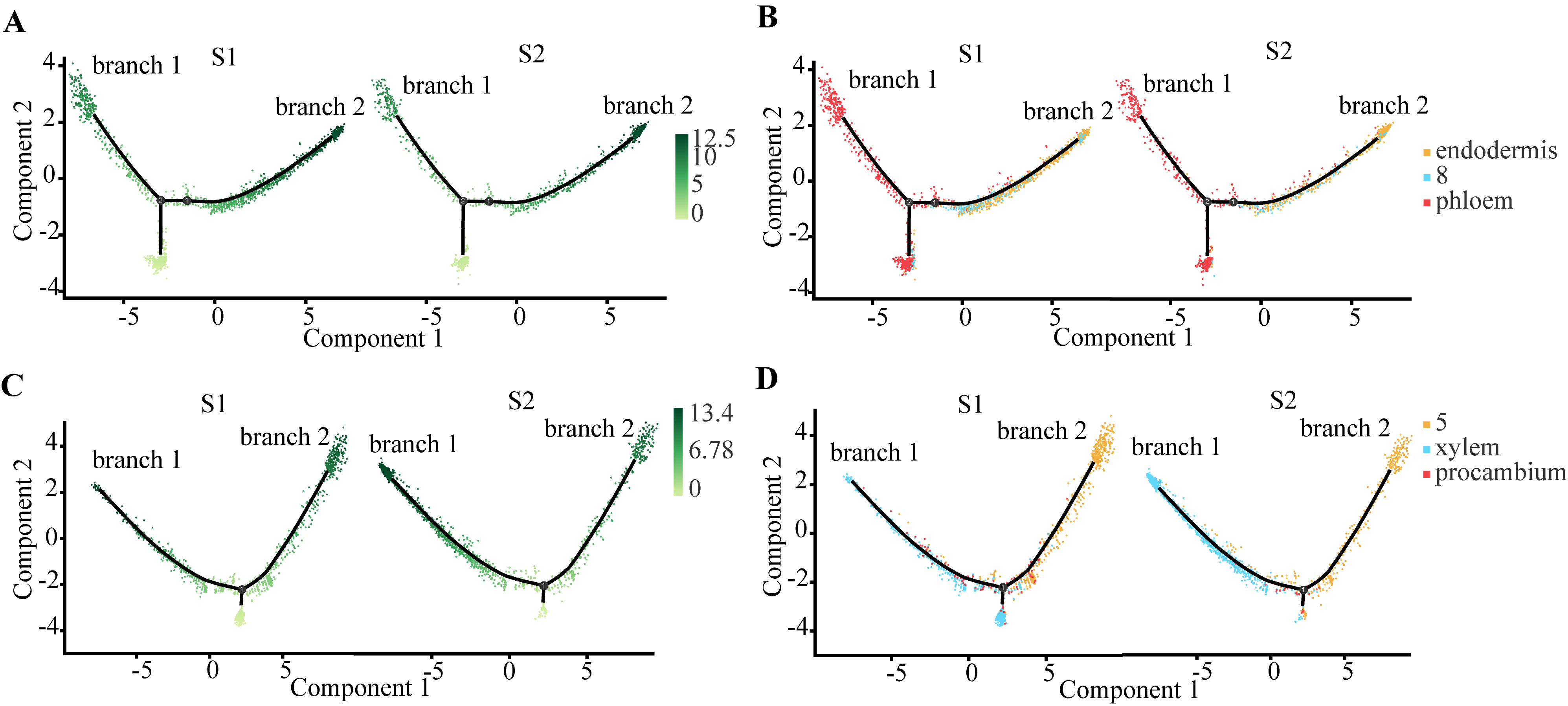

Supplement: Supplementary Figure 1 — Identification of Cluster 5. (A) Single-cell Transcriptome data of endodermis, cluster 8 and phloem from S1 and S2 analyzed by Monocle 2, revealing the differentiation trajectory of them. Each dot indicates a single cell. Different color on the dots denotes the pseudotime score. (B) Cell types labeled on the differentiation trajectory for S1 and S2, respectively. (C) Single-cell Transcriptome data of xylem, cluster 5 and procambium from S1 and S2 analyzed by Monocle 2, revealing the differentiation trajectory of them. Each dot indicates a single cell. Color on the dots indicates the pseudotime score. (D) Cell types labeled on the differentiation trajectory of S1 and S2, respectively. [file Image_1.tif]

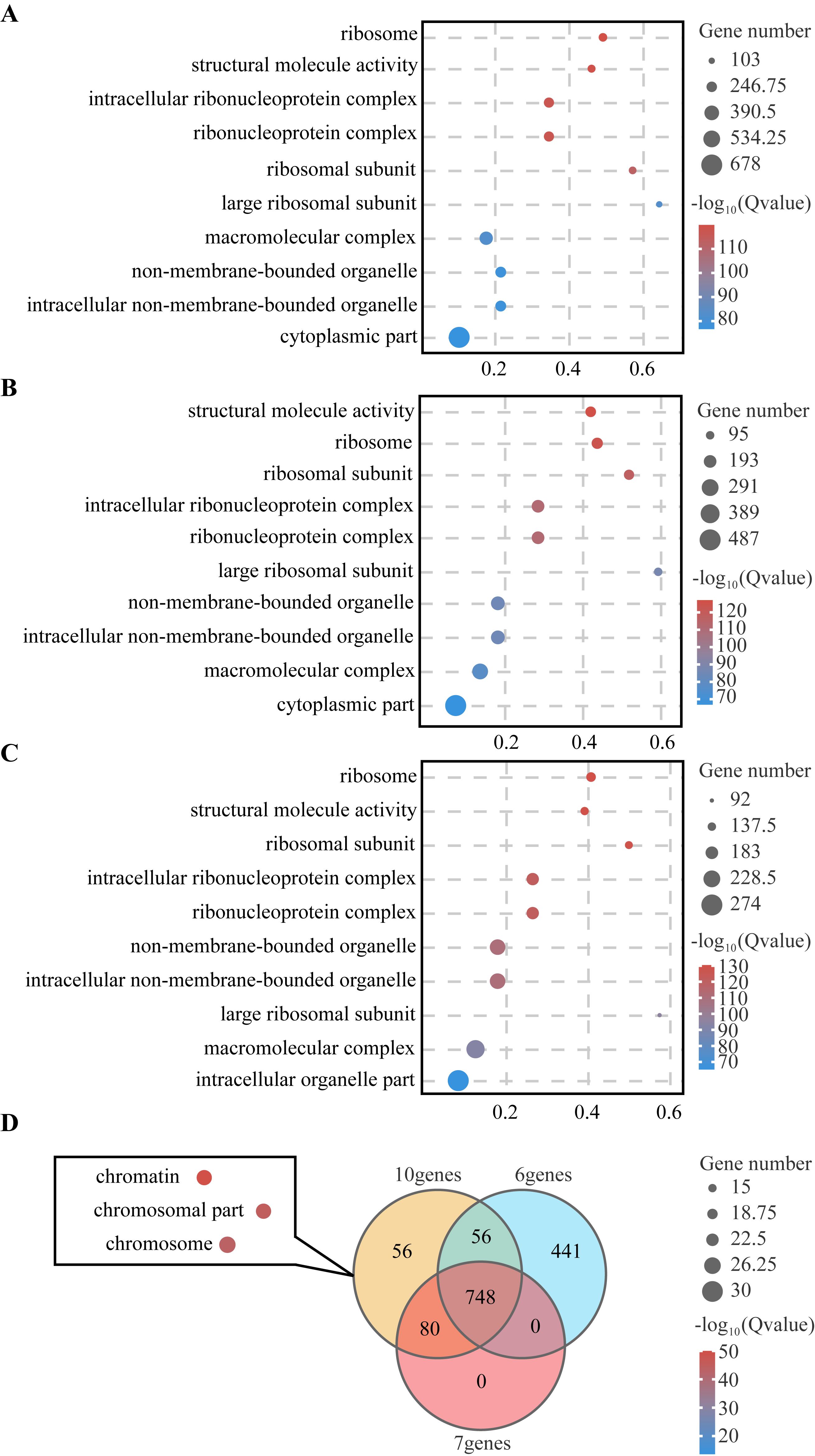

Supplement: Supplementary Figure 2 — Identification of Cluster 10. (A–C) Scatter plots of GO enrichment analysis of up-regulated genes for cluster 6 (A), cluster 7 (B) and cluster 10 (C) cell types after the aggregation of S1 and S2. (D) Scatter plots of GO enrichment analysis of 56 up-regulated genes specific to cluster 10 cell types after the aggregation of S1 and S2. [file Image_2.tif]

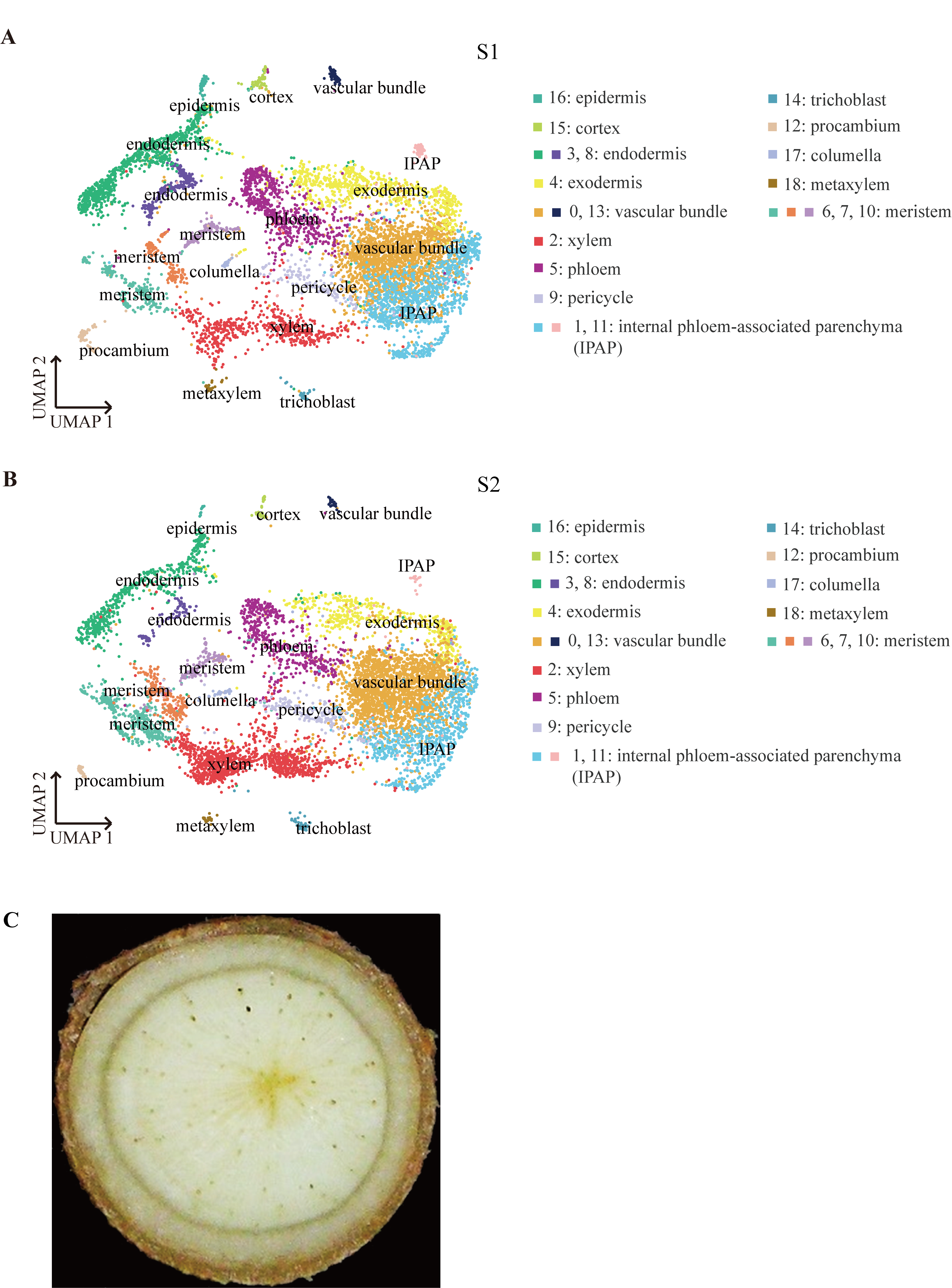

Supplement: Supplementary Figure 3 — The Umap visualization of each cluster in the cassava tuberous root of S1 and S2, respectively. (A) Umap visualization of each cluster in the cassava tuberous root of S1 stage. Each dot indicates a single cell. Each color indicates a cell cluster. (B) Umap visualization of each cluster in the cassava tuberous root of S2 stage. Each dot indicates a single cell. Each color indicates a cell cluster. (C) The cross section of the Cassava tuberous root. [file Image_3.tif]
